# An Investigation into Some Effective Factors on Encapsulation Efficiency of Alpha-Tocopherol in MLVs and the Release Profile from the Corresponding Liposomal Gel

**Published:** 2013

**Authors:** Hosseinali Tabandeh, Seyed Alireza Mortazavi

**Affiliations:** *Department of Pharmaceutics, School of Pharmacy, Shahid Beheshti University of Medical Sciences, Vali-e-Asr Ave., Niayesh Junction, P.O. Box 14155-6153, Tehran, Iran.*

**Keywords:** Liposome, *α*-tocopherol, Cholesterol, Liposomal gel, Release, Encapsulation efficiency

## Abstract

Vitamin E (*α*-tocopherol) is a natural antioxidant very useful for preventing the harmful effects of UV sun rays as skin aging and cancers. In this study, different MLV formulations were made using egg lecithin and varying molar ratios of *α*-tocopherol and/or cholesterol, and their encapsulation efficiencies were determined. The best liposomal product was incorporated into a carbomer 980 gel. The resulting preparation was then studied with regard to the rheology and release profile using r^2^ values and Korsmeyer-Peppas equation. The encapsulation efficiency was dramatically decreased when using *α*-tocopherol at molar ratios of 1:10 or more, which is suggested to be due to the defect in regular linear structure of the bilayer membrane. Addition of cholesterol to formulations caused a decrease in encapsulation efficiency directly related to its molar ratio, which is due to the condensation of the bilayer membrane as well as competition of cholesterol with *α*-tocopherol. The liposomal gel showed a yield value of 78.5 ± 1.8 Pa and a plastic viscosity of 27.35 ± 2.3 cp. The release showed a two-phase pattern with the zero-order model being the best fitted model for the first phase. However, the “n” and r^2 ^values suggested a minor contribution of Higuchi model due to some diffusion of *α*-tocopherol from the outermost bilayers of the MLVs to the gel. The second phase showed a non-Fickian release indicating a more prominent role for diffusion. This combinational release profile provides a high initial concentration of *α*-tocopherol followed by a slow release throughout a 10 h period.

## Introduction

The free radicals produced by the UV sun rays cause destruction of the DNA in skin cells and also an increase in disruption rate of the matrix proteins of the skin. The natural anti-oxidant vitamin E (*α* tocopherol) due to its anti-oxidant action could prevent the DNA damage in skin triggered by UV rays (1, 2). Its good anti-oxidant effect on skin as well as lacking any notable side effect has made *α*-tocopherol a useful ingredient in combination with sun-screens in cosmetic products intended for sun protection (3-6).

From cosmetic point of view, it could also have potential benefits in prevention of the skin aging.

Liposomes are phospholipid bilayer vesicles which were first introduced and described by Bangham *et al*. (7, 8). Due to the specific structure and characteristics, liposome ,it was considered as a potential candidate to encapsulate drugs and enzymes and act as a novel drug delivery system (9, 10). Since then, many potential benefits have been found for them as a drug delivery system for targeting and controlling the release in various fields such as delivery of antineoplastic drugs, peptides and proteins, antibiotics, and cardiovascular targeting; thus, they have become the focus of various fields of pharmaceutical research (11-18). Liposomes were also found to have various trans-cellular, inter-cellular, and follicular ways of penetration into the skin on topical application, which opened up a new horizon in the field of topical delivery of drugs, genes, proteins and peptides for conquering the absorption obstacles, targeting and better efficacy (19). Besides, liposomes could insert their phospholipids into the inter-cellular lipid structure of the skin and make changes which affect the hydration status, elasticity, and barrier effect (20-23). This could explain the refreshing effect of even the empty liposomes. Therefore, liposomes have also found increasing importance in the field of cosmetics (24-27).

Liposomal form of *α*-tocopherol has been shown to penetrate more into the stratum corneum and caused a depot action which makes it more efficient in exerting the antioxidant action for skin (5, 28). Considering the antioxidant activity of *α*-tocopherol which prevents the harmful effects of UV rays on DNA and matrix proteins of skin, efficient topical delivery of *α*-tocopherol is beneficial for both medical and cosmetic purposes. In formulating topical liposomal forms, the best semi-solid base is the gel form both from pharmaceutical point of view and the consumer’s better feeling (29).

The present study was aimed toward formulating a liposomal gel of *α*-tocopherol for topical application. Due to the lipophilic nature of *α*-tocopherol and its incorporation into the bilayer membrane of liposomes, the effects of molar ratio of *α*-tocopherol (to the phospholipid) on the encapsulation efficiency was investigated first. Cholesterol is an important ingredient usually used in liposome formulation to give a better stability and slower release of the encapsulated active agents. However, since it is incorporated into the liposomal bilayer, it will have interaction with encapsulation of the lipophilic substances such as *α*-tocopherol. Therefore, the effect of cholesterol on encapsulation efficiency was also investigated in this study. Different carbomers were then investigated to select the best gel base for incorporation of the liposomal preparation. Then, the rheology and release profile of the liposomal gel was studied.

## Experimental

Egg phosphatidylcholine (Ovotin 160^®^) was gifted by Faratin Company, Tehran, Iran (representative of Lucas Meyer, Germany). DL- *α*-tocopherol was obtained from DSM Company, Switzerland. The three acrylic acid polymers, *i.e. *carbomers 934, 940, and 980 were from Noveon, USA. Chloroform, Cholesterol, Triton X100 and other materials were all from Merck, Germany. Dialysis Bags were purchased from Sigma-Aldrich, Germany (D 9777 with a cut-off of 12400).

*Preparation of α-tocopherol MLVs*

Multilamellar Large Vesicles (MLVs) were prepared by the classic Bangham hydration method (8). Different formulations were prepared with 66 μmole of egg lecithin (Ovotin 160^®^) along with different molar ratios of *α*-tocopherol to egg lecithin (*i.e. *1:33, 1:25, 1:20, 1:10, 1:5, 1:3.3, 1:1.25, and 1:1). They were dissolved in chloroform and rotary evaporated at 45ºC under reduced pressure. The resulted films were then hydrated by a standard PBS solution with a pH of 7.45 during a 30 min period. Each formulation was prepared three times.

*Preparation of α-tocopherol MLVs containing various molar ratios of cholesterol*

Each series of MLVs containing 1:33, 1:25, 1:20, 1:10, and 1:5 molar ratios of *α*-tocopherol to lecithin were prepared similar to the previously mentioned method but with cholesterol at molar ratios of 1:20, 1:10, and 1:1 to lecithin.

*UV absorption spectrum and calibration curve of α-tocopherol*

The UV absorption spectrum of *α-*tocopherol from a 10 μg/mL solution was obtained to find the peak wavelength. The calibration curve was then plotted by determination of the absorption of different concentrations of α-tocopherol in chloroform (concentrations of 0.0500, 0.0750, 0.1000, 0.1250, 0.1500, and 0.1750 mg/mL) at the peak wavelength of 287.5 nm.

*Determination of the α-tocopherol encapsulation efficiencies in different series of MLVs*

The liposomal suspensions were centrifuged at 5000 rpm (274 g). The resulted concentrated

liposomal pellets were washed twice with the PBS solution, after which they were dissolved in

Triton X100 solution. In cholesterol containing formulations, they were again centrifuged to separate the undissolved cholesterol (30).

The *α*-tocopherol concentrations were then determined spectrophotometrically at 287.5 nm, and the encapsulation efficiencies were calculated.

*Formulation of the base gel for liposome incorporation*

Different series of gels using carbomers 934, 940, and 980 at different concentrations of 0.1, 0.3, and 0.5% were prepared by gently dispersing the required amount of the carbomer in a mixture of water and glycerin, and giving them 2 h for hydration. Then, pH was adjusted to 5-5.5 with a 0.1 N solution of NaOH to form the gels.

*Quality Control of the gels*

The gels were evaluated with regard to the apparent consistency and quality of the film formed after drying of the gel. Three samples from each formulation were placed in refrigerator and three samples at 40ºC. The samples underwent a heat-cold cycle by replacing them at 24 h intervals. After ten days, the samples were evaluated again.

*Formulation of the α-tocopherol liposomal gel*

Required amount of the selected liposomal preparation of *α*-tocopherol was added to the selected base gel to obtain an ultimate concentration of 10% w/w of the liposomal suspension in gel, and mixed thoroughly to reach a uniform gel.

*Assay of α-tocopherol in the liposomal gel*

A specified amount of the liposomal gel was diluted with an appropriate amount of the PBS solution and mixed thoroughly. Then, Triton X100 was added to the mixture and mixed for 1 h, after which the mixture underwent centrifugation at 5000 rpm (274 g). The supernatant was assayed spectrophotometrically.

*Rheologic studies*

One mL of the liposomal gel was placed into the plate of the “Brookfield DV III LV Ultra” viscometer and by using the spindle no. 52 at the speeds of 0.3 to 100 rpm, the shear rates and shear stresses were reported and the rheograms were printed.

*Studying the α-tocopherol release profile from the liposomal gel*

A 1.5 g sample of the liposomal gel was placed as a uniform and thin film on the dialysis membrane in the upper section of the diffusion cell (as the donor phase) at 37ºC. The diffusion cell had a volume of 25 mL and a diffusion surface area of 5 cm^2^ (31). After predetermined intervals until 24 h, 2 mL samples were collected from the acceptor phase and were replaced with 2 mL of the fresh acceptor phase (PBS solution). The samples were analyzed spectrophotometrically at 287.5 nm. The cumulative percentages of the *α*-tocopherol released were plotted against time. The data were fitted in Higuchi, zero-order, and first-order kinetic models and the r^2^ (coefficient of determination) values were determined. The data were also placed in the Korsmeyer-Peppas power law equation to obtain the n-values.

## Results and Discussion

The formulations’ components and the encapsulation efficiencies for the first series of MLVs made with different molar ratios of *α*-tocopherol to phospholipid are shown in Table 1.

**Table 1:** Encapsulation efficiencies of formulations with varying molar ratios of *α*-tocopherol

**Formulation**	***α*** **-tocopherol molar ratio**	**E. E. (%)**
**L-1**	1:33	98.6 ± 1.6
**L-2**	1:25	90.1 ± 2.4
**L-3**	1:20	93.4 ± 2.3
**L-4**	1:10	50.3 ± 1.2
**L-5**	1:5	28.0 ± 0.8
**L-6**	1:3.3	19.5 ± 0.6
**L-7**	1:1.25	11.9 ± 0.5
**L-8**	1:1	9.7 ± 0.2

 As it is shown, in formulations containing 1:33 to 1:20 molar ratios of *α-*tocopherol, the encapsulation efficiencies remained high (higher than 90%), but at molar ratio of 1:10 the encapsulation efficiency was decreased significantly (p < 0.05). At molar ratios of 1:5 and more, the encapsulation efficiency showed a dramatic decrease. Due to the lipophilic nature of *α*-tocopherol molecule, it is incorporated into the liposomal bilayer between the lipophilic chains of the phospholipid molecules. At low molar ratios, it could completely be incorporated into the bilayer membrane, which explains the high encapsulation efficiencies observed in the present study at low molar ratios. Furthermore, in a previous work, a high encapsulation efficiency of 98-101% for *α*-tocopherol has been reported (5). In low molar ratios under 10% (or 1:10), *α*-tocopherol is stoichiometrically incorporated into the membrane in monomeric form, which results in condensation and a decrease in crosssectional surface area of the membrane. However, at higher molar ratios *α*-tocopherol-rich regions with high and low frequencies are formed which causes ripple phases, and extensive increase in its molar ratio results in destruction of the regular bilayer structure (32, 33). Considering the initial amounts of *α*-tocopherol used in formulations and the encapsulation efficiencies observed, the amount of *α*-tocopherol encapsulated showed an increase up to 1:20 molar ratio (*i.e*. 5%), after which the amounts were relatively constant while the encapsulation efficiencies decreased. Such a trend has previously been reported for another lipophilic material to be encapsulated, *i.e*. nevirapine (34). In that study, the same trend was observed but with 20% (1:5) ratio being the determining point after which the encapsulation efficiency decreased. This difference in the determining point is suggested to be due to the difference in molecular structure and the capability of incorporation into the bilayer membrane. However, in a previous study on the lipophilic drug benzocaine, it was reported that by increasing the amount of the initial drug added to the lipid phase for liposome formation, pure amounts of the encapsulated drug increased while the encapsulation efficiencies remained relatively constant at about 30% (35). This difference with our results is speculated to be due to the different solubility of benzocaine in the liposomal bilayer and the higher ratios used, which resulted in lower encapsulation efficiencies observed. Considering the observed results and the fact that the antioxidant action of *α*-tocopherol is exerted by its monomeric form, 1:20 (5%) molar ratio of *α*-tocopherol was selected as the optimum ratio for formulation of the liposomes. As it was previously mentioned, each series containing 1:33, 1:25, 1:20, 1:10, and 1:5 (3, 4, 5, 10, and 20% respectively) molar ratio of *α*-tocopherol to lecithin were separately formulated in three different formulations containing 1:20, 1:10, and 1:1 (5, 10, and 100% respectively) molar ratios of cholesterol. The formulations’ components and the encapsulation efficiencies for the MLVs made with different molar ratios of cholesterol to the phospholipid are shown in Table 2.

**Table 2:** Encapsulation efficiencies of formulations with varying molar ratios of *α*-tocopherol and cholesterol**.**

**Formulation**	***α*** **-tocopherol molar ratio**	**Cholesterol molar ratio**	**E. E. (%)**
**L-9**	1:33	1:20	93.3 ± 1.2
**L-10**	1:25	1:20	89.1 ± 0.8
**L-11**	1:20	1:20	86.1 ± 1.1
**L-12**	1:10	1:20	46.5 ± 0.6
**L-13**	1:5	1:20	26.6 ± 0.5
**L-14**	1:33	1:10	90.4 ± 1.1
**L-15**	1:25	1:10	85.5 ± 1.3
**L-16**	1:20	1:10	74.0 ± 0.7
**L-17**	1:10	1:10	40.8 ± 0.9
**L-18**	1:5	1:10	22.9 ± 0.8
**L-19**	1:33	1:1	57.1 ± 2.2
**L-20**	1:25	1:1	49.9 ± 2.5
**L-21**	1:20	1:1	45.7 ± 1.9
**L-22**	1:10	1:1	24.2 ± 1.8
**L-23**	1:5	1:1	13.8 ± 1.3

 It was observed that at each level of *α*-tocopherol, addition of cholesterol caused a minor but significant (p < 0.05) decrease in encapsulation efficiency which was directly related to the amount of cholesterol.

Similar results have previously been reported for some lipophilic drugs as triamcinolone acetonide (36), clotrimazole (37), ciprofloxacin (38), dexamethasone (39), ibuprofen and diazepam (40). Cholesterol molecules are placed between the adjacent phospholipid molecules in liposomal bilayer and hence occupy some space and compete with *α*-tocopherol for incorporation into the bilayer. Additionally, cholesterol makes the bilayer more rigid, which makes the incorporation of the *α*-tocopherol molecules harder. In this study, 1:1 molar ratio of cholesterol to the phospholipid caused a dramatic decrease in encapsulation efficiency, which is speculated to be due to defect in the regular linear structure of the liposomal bilayer (41). Disruption of the regular linear structure of liposomal bilayer causes a prominent decrease in encapsulation efficiency for both lipophilic and hydrophilic molecules (38, 41). A recent study on vitamin E (*α*-tocopherol) reported no difference in the amount of encapsulated *α*-tocopherol in presence of cholesterol at a molar ratio of 3:10 to the phospholipid (42). This study did not report the encapsulation efficiency; instead, the total amount of *α*-tocopherol encapsulated in liposomes with a single molar ratio of cholesterol was compared to that of the liposomes without cholesterol. Also, different molar ratio of the *α*-tocopherol was not studied. Considering the minor effect observed in our study, the difference could be due to the difference in preparation conditions. On the contrary, some previous studies on liposomes encapsulating the lipophilic drugs enrofloxacin (43), ibuprofen (44) and albandazole (45) reported that the presence of cholesterol could increase the encapsulation efficiency, and related this effect to more close binding of the phospholipid molecules and the higher rigidity of liposomal membrane. It is also likely that the difference in molecular structure of these drugs with α-tocopherol studied here resulted in the different observed trend in encapsulation efficiency by addition of cholesterol. It is speculated that in these instances, the cholesterol molecule does not occupy the specific space in which the drug is placed inside the bilayer membrane; thus it could make the membrane more rigid for keeping the encapsulated drug inside the membrane without interfering with the needed space inside the bilayer membrane.

 A potential benefit of cholesterol in liposomes is that although the encapsulation efficiency could be decreased, the escape or release of drug molecules from liposomal membrane could also be decreased due to an increase in membrane rigidity, which has previously been reported for liposomal triamcinolone acetonide (36). Therefore, among the different molar ratios of cholesterol investigated in this study, 1:20 molar ratio was selected as the optimum formulation. For topical application of liposomes, the best dosage form is gel form both from pharmaceutical point of view and the consumer’s better feeling (29). Among the gelling polymers used for this purpose, carbomers have been extensively used for their bioadhesivity, compatibility with liposomes, increasing the stability, and sustaining the release compared to plain liposomes (37, 38, 46, and 47). In this study, three concentrations of 0.1, 0.3, and 0.5% from three carbomers 934, 940, and 980 were used to prepare the gels.Carbomer 934 and 940 formulations did not show suitable characteristics in any of the three concentrations. However, the 0.5% carbomer 980 gel showed desirable characteristics with regard to appearance, consistency, spreadability, and maintaining the desirable quality after the heatcold cycles. Therefore, it was selected as the base formulation for incorporation of the liposomes.

After incorporation of the liposomes into the selected base gel, the resulting liposomal gel was assayed and showed a result of 97.9±0.8% with respect to *α*-tocopherol.

**Figure 1:**
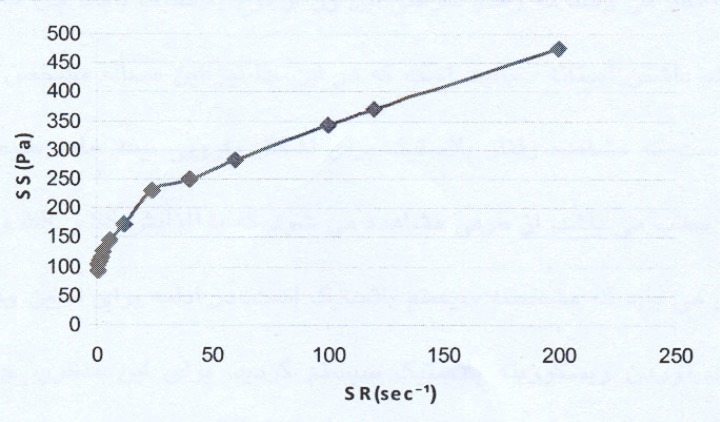
Rheogram of the *α*-tocopherol liposomal gel.SR: Shear Rate; SS: Shear Stress

The rheogram of the selected liposomal gel is shown in Figure 1. 

As the rheogram indicates, the system shows a plastic behavior with a yield value. Since low shear rates, *i.e. *lower than 5 sec^-1^, were used (the study started with the shear rates as low as 0.6 sec^-1^), the result indicating a yield value is completely reliable.

**Figure 2:**
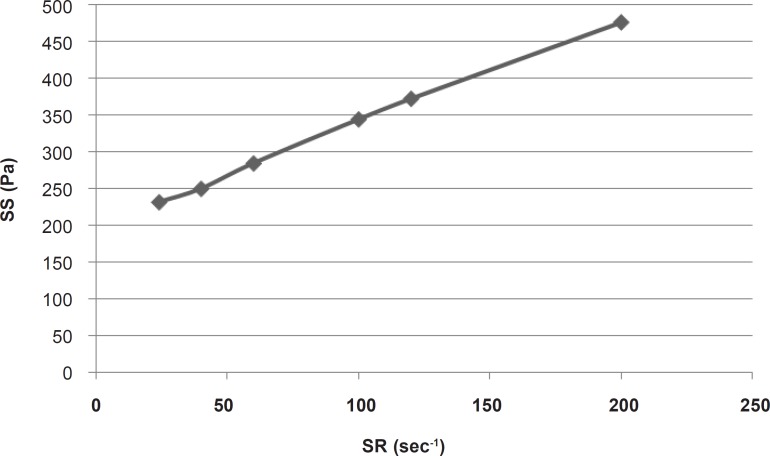
Linear part of the rheogram of *α*-tocopherol liposomal gel. SR : Shear Rate; SS: Shear Stress.

For calculating the plastic viscosity and yield value, the first points in nonlinear portion of the rheogram were omitted consequently until reaching the linear part which was in the range of 24 sec^-1^ to 200 sec^-1^ (Figure 2).

**Figure 3:**
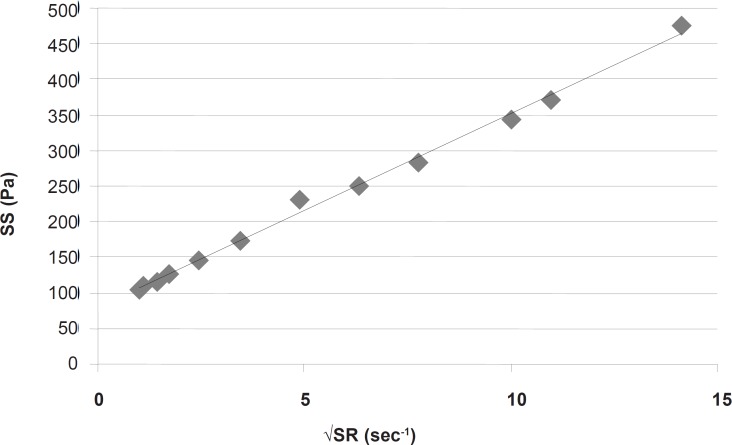
Fitch model of the rheogram of *α*-tocopherol liposomal gel. √SR: Square Root of Shear Rate; SS: Shear Stress.

This part had a coefficient of determination (r^2^) equal to 0.997 which confirmed the linearity. The slope indicated a plastic viscosity of 1.40±0.12 cp. The intersection of the extrapolated linear part with the shear stress axis showed a Bingham yield value of 198/7 ± 0.8 Pa. For better and more reliable calculation, Casson or Fitch models should be used. In Casson model, the second root of shear stress is plotted against the second root of shear rate, while in Fitch model the shear stress is plotted against the second root of shear rate. The r^2^ values for the Fitch and Casson models were 0.996 and 0.986 respectively. Therefore, the Fitch model (Figure 3) was used to calculate the yield value and plastic viscosity being 78.5 ± 1.8 Pa and 27.35 ± 2.33 cp respectively. 

Both values were significantly different (p < 0.05) from the previous results obtained by omitting the points in nonlinear part of the rheogram. This was predictable since after reaching the yield value, although the gel begins to flow but the gel structure resists and the viscosity is higher than that in higher shear rates, and the slope is changing with increasing shear rate.

**Figure 4:**
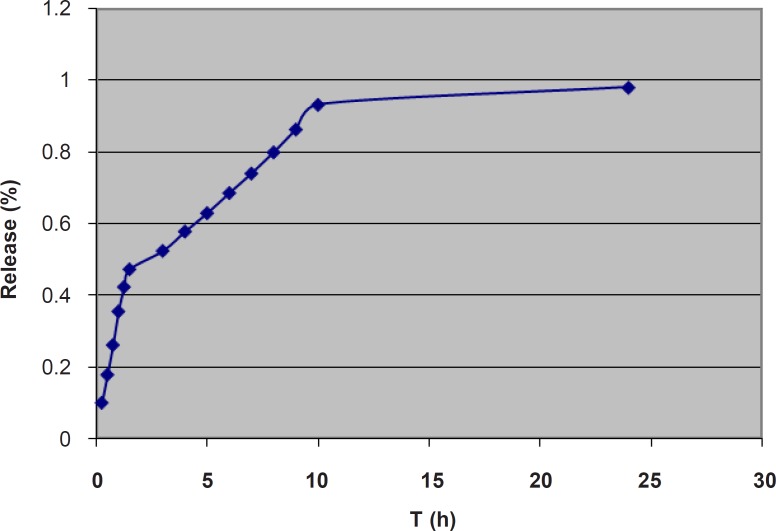
Cumulative percent release of α-tocopherol from the liposomal gel. T (h): Time in h; Release (%): Cumulative percent release.

 For studying the release process, the cumulative percentages of the *α*-tocopherol released were plotted against time (Figure 4).

The data were fitted in Higuchi, zero-order, and first order kinetic models and the r^2^ (coefficient of determination) values were determined. The data were also fitted in the Korsmeyer-Peppas power law equation to obtain the n-values. The obtained kinetic parameters are shown in Table 3.

**Table 3:** Kinetic parameters for the release process

	**Zero-order**	**Higuchi**	**First-order**	**Korsmeyer-Peppas**	
	r^2^	r^2^	r^2^	r^2^	n
**Whole process**	0.675	0.890	0.330	0.905	0.467
**First stage**	0.993	0.990	0.957	0.994	0.913
**Second stage**	0.995	0.998	0.990	0.991	0.776

Considering the whole process of release, neither of the r^2^ values for the Higuchi, zero-order and

first-order models were near 1, the highest value being 0.890 for the Higuchi model. However, the release graph (Figure 4) implies a three-phase release pattern, the third phase being negligible. Therefore, the release process could be considered as a two-phase. The kinetic parameters for the first part are also shown in Table 3. The highest r^2^ value was 0.993 for zero-order model, which implies a zero-order model of release for the first stage. The n-value of 0.913 also confirms the zero-order model as the best fitted model. This is speculated to be mainly due to the unencapsulated *α*-tocopherol dispersed through the gel. However, the slight negative deviation from 1, as well as the relatively high value of r^2 ^for Higuchi model implies some contribution of the diffusion mechanism and Higuchi model in the process. This is suggested to be related to diffusion of the encapsulated α-tocopherol from the outermost bilayers of MLVs to the gel. Progression of the release process would gradually deplete the gel and the outer bilayers of MLVs from *α*-tocopherol and the release would more depend on the inner bilayers. The kinetic parameters for the second part are also shown in Table 3. In the second stage, the highest r^2^ value was 0.998 for the Higuchi model, which implies Higuchi model as the best fitted model for the second stage. However, the relatively high value of r^2^ for zero-order model as well as the n-value of 0.776 implies some contribution of zero-order model in the release process. It is speculated that in this stage, since most of the *α*-tocopherol present in gel and the outer bilayers has been released, the encapsulated *α*-tocopherol from the inner bilayers finds a more prominent role and the release rate is also decreased. In a previous study on the lipophilic drug Griseofulvin, n-values between 0.5 and 1, and concomitant contribution of Higuchi and zero-order models were reported and it was concluded that the diffusion of the unencapsulated drug through the gel was much faster than the release rate from the liposomal membrane which acted as a reservoir (48). On the other hand, the reverse trend has previously been reported for two hydrophilic drugs. A previous study on release of 5-flurouracil from liposomal chitosan hydrogels, reported a mainly Higuchi model and diffusion-controlled release pattern for the first 1.5 h, after which the zero-order model had found a more important role in the release process (49). Also, in a study on release of the hydrophilic drug lidocaine HCl from liposomal carbomer gel, the release pattern was reported to follow Higuchi model for the first 3 h, and zero-order model for the rest (47). It seems that in those studies, the first part indicated the main contribution by diffusion of the unencapsulated drug through the gel, after which the rate limiting step of passage of the hydrophilic drug through the liposomal membrane found a more prominent role.

## Conclusion

At low molar ratios (lower than 1:10), *α*-tocopherol is present in its monomeric form inside the liposomal membranes. Considering the observed results and the fact that the antioxidant action of *α*-tocopherol is exerted by its monomeric form, 1:20 molar ratio of *α*-tocopherol was selected as the optimum amount for formulation of the liposomes to have the highest encapsulated amount in the active form. The *α* tocopherol liposomal gel showed a two-phase release pattern with a faster zeroorder release as the first stage, followed by a slower diffusion-based Higuchi model release. This release profile provides a high initial dose followed by a slow-release maintenance dose over a 10-h period.
